# Editorial

**DOI:** 10.22336/rjo.2022.52

**Published:** 2022

**Authors:** Mihail Zemba

**Affiliations:** * Editor in Chief

My collaboration with the journal “Oftalmologia” dates back to 1994, when I started my residency in Ophthalmology. I began by helping my supervisor at that time, Ioan Ştefăniu, MD, PhD, with the distribution of the journals. In 1996, I published my first article and in 1997, I became editor.

I was taught about the structure and functioning of a medical journal by my predecessors, Prof. Paul Cernea, Assoc. Prof. Mircea Filip and Ioan Ştefăniu, MD, PhD. Under their supervision, “Oftalmologia” evolved to become an important name among the medical journals in Romania. 

To increase the international exposure of the scientific works published in “Oftalmologia”, at the initiative of Prof. Călin Tătaru, MD, PhD, in 2015, the journal started to publish articles in English and changed its name to “Romanian Journal of Ophthalmology”. Simultaneously, the online, free access version of the journal was launched, on www.rjo.ro. 

Despite a slower start, Romanian Journal of Ophthalmology has become internationally known, as it results from the more than 3000 monthly views from all around the world, as well as from the increasing number of citations of published articles.

Authors from more than 25 countries from Europe, Asia, Africa, and Latin America have published their works in Romanian Journal of Ophthalmology over the years. Regrettably, the contribution of Romanian authors has decreased in the last years and there are issues of the journal in which more than 80% of the scientific work belongs to foreign authors. 

This year, we started the process of indexing the journal in Clarivate - Web of Science (ISI) database, which is still ongoing.

To progress, every organization benefits from the periodical change of its managing team. This avoids routine and allows new ideas and perspectives to spring and drive the journal forward. I am confident that the new board of Romanian Journal of Ophthalmology will ensure its growth, enhancing its visibility and prestige. 


**Mihail Zemba, MD, PhD,**
**Editor in Chief**
**Romanian Journal of Ophthalmology**

